# Adsorption of Diffusing Tracers, Apparent Tortuosity, and Application to Mesoporous Silica

**DOI:** 10.1021/acs.langmuir.3c03855

**Published:** 2024-05-17

**Authors:** Nathann Teixeira Rodrigues, Fábio David Alves Aarão Reis

**Affiliations:** Instituto de Física, Universidade Federal Fluminense, Avenida Litorânea s/n, 24210-340 Niterói, RJ, Brazil

## Abstract

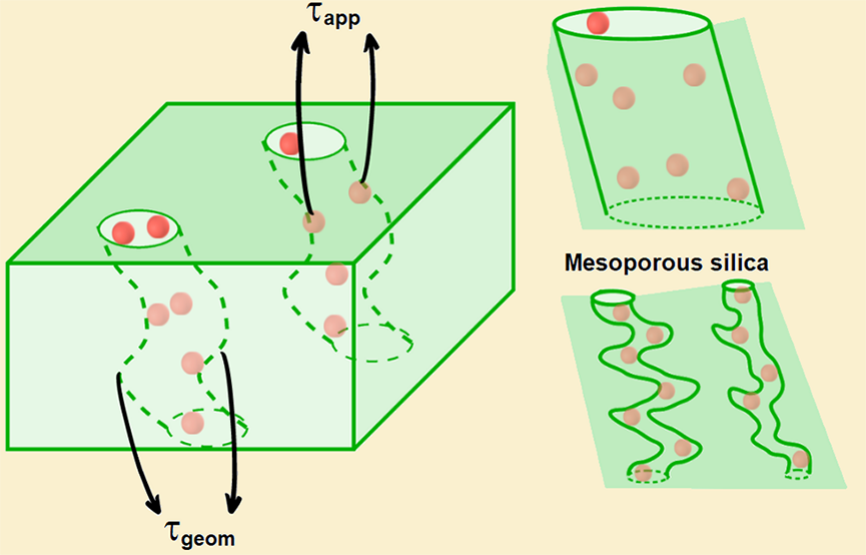

The apparent tortuosity due to adsorption of diffusing tracers in a porous material is determined by a scaling approach and is used to analyze recent data on LiCl and alkane diffusion in mesoporous silica. The slope of the adsorption isotherm at small loadings is written as β = *q*_*A*_/*q*_*G*_, where *q*_*A*_ is the adsorption–desorption ratio and *q*_*G*_ = ϵ/(*as*) – 1 is a geometrical factor depending on the range *a* of the tracer-wall interaction, the porosity ϵ, and the specific surface area *s*. The adsorption leads to a decrease of effective diffusion coefficient, which is quantified by multiplying the geometrical tortuosity factor τ_*geom*_ by an apparent tortuosity factor τ_*app*_. In wide pores or when the adsorption barrier is high, τ_*app*_ = β + 1, as obtained in previous works, but in narrow pores there is an additional contribution from frequent adsorption–desorption transitions. These results are obtained in media with parallel pores of constant cross sections, where the ratio between the effective pore width ϵ/*s* and the actual width is ≈0.25. Applications to mesoporous silica samples are justified by the small deviations from this ideal ratio. In the analysis of alkane self-diffusion data, the fractions of adsorbed molecules predicted in a recent theoretical work are used to estimate τ_*geom*_ of the silica samples, which is ≫1 only in the sample with the narrowest pores (nominal 3 nm). The application of the model to Li^+^ ion diffusion leads to similar values of τ_*geom*_ and to a difference of energy barriers of desorption and adsorption for those ions of ∼0.06 eV. Comparatively, alkane self-diffusion provides the correct order of magnitude of τ_*geom*_, with adsorption playing a less important role, whereas adsorption effects on Li^+^ diffusion are much more important.

## Introduction

The diffusion of molecules, nanoparticles, or colloidal particles in porous materials has applications in several areas,^1−5^ such as heterogeneous catalysis,^6,7^ membrane separation or distillation,^8,9^ gas exploration,^10^ and environmental remediation.^11,12^ The confinement of a tracer in a porous medium usually leads to a decrease of the effective diffusion coefficient *D*_*ef*_ relatively to the bulk coefficient *D*_*B*_ (measured in nonconfined conditions), which may be quantified by splitting the confinement effects into two contributions: first, the porosity ϵ (defined as the ratio between pore volume and total volume of a sample) reduces the volume available for the tracer movement; second, a lumped parameter called tortuosity (or tortuosity factor),^1−5^ here denoted as τ, represents other possible mechanisms that affect the tracer movement. *D*_*ef*_ is then written as
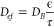
1

In systems with only hard core interactions between tracers and wide pores, τ accounts for the tortuous geometry of the pore network, which explains the origin of this term. However, some tracers may not be able to cross the constrictions of a pore network, so hindrance mechanisms also affect the values of τ obtained from 1.^13−16^ In extreme but not infrequent cases, *D*_*ef*_ may exhibit a dependence with the time scale or with the length scale of observation, so the diffusion becomes anomalous.^17−20^

There are additional delays in the tracer displacement if they are adsorbed at the pore walls during a significant fraction of their diffusion time,^6,21−25^ as in a stop-and-go process.^26^ Thus, τ may be written as the product of an apparent tortuosity τ_*app*_ related to the adsorption–desorption transitions and a geometrical tortuosity τ_*geom*_ related to the tracer and medium geometries, i.e. encompassing the intrinsic tortuosity of the network and hindrance effects. Previous works proposed to represent the former effects by writing τ_*app*_ = β + 1, where β is the slope of the adsorption isotherm at small loadings.^10,27,28^ This standard formula directly follows from the partitioning of tracers in surface and bulk regions and the assumption that adsorbed tracers are immobile.

The first aim of our work is to determine the effective diffusion coefficient in molecular diffusion processes in media whose pores may be narrow or large depending on the relation between their widths and the ranges of the tracer-wall interactions. Within this approach, the standard formula for the apparent tortuosity is applicable for wide pores, but we show that adsorption–desorption transitions have non-negligible contributions to the mean square displacements (MSDs) in narrow pores and when adsorption barriers are low. We also advance in showing how the slope β can be written in terms of those transitions’ rates and of the medium geometry (porosity and specific surface area), which allows its estimation from physical and chemical parameters without the direct measurement of the isotherm. Our calculation of the MSD is exact for membranes with parallel pores of constant width, but the small differences in the relevant geometrical factors in media with tortuous and intersecting pores suggest a broader applicability of the results. Expected deviations due to steric hindrance are also discussed.

The second aim of our work is to use this approach to analyze recent data on diffusion of inorganic and organic molecules in mesoporous silica.^29,30^ This is a class of materials with high surface area and high porosity, with applications in catalysis,^31^ retention of environmental pollutants,^32^ drug delivery,^33^ and energy storage,^34^ which explains the large interest in understanding the transport of fluids and solutes in their pores. Our treatment considers the experimentally measured values of porosity, surface area, and tortuosity [from Eq. 1 with different tracers], combined with expected ranges of microscopic physicochemical parameters, to estimate the geometrical tortuosities of the silica samples. The difference between activation energies of desorption and adsorption of Li^+^ is obtained and the overall results indicate that alkane self-diffusion provides the correct order of magnitude of the geometrical tortuosity even in samples with very narrow pores.

The paper is organized as follows. First, we present details of the model and the method of solution along with the quantities of interest. The solution of the model, discussion of its main features, the application to the diffusion in mesoporous silica, and relations with other works are then presented. Lastly, we present our conclusions. A list of the symbols used in this work can be found in Table 1.

**Table 1:** List of Symbols Used on This Work

Symbol	Quantity	Physical dimension
*a*	Width of surface (S) region	*L*
*a*_*A*_^2^	Adsorption mean square displacement (*x*)	*L*^2^
*a*_*D*_^2^	Desorption mean square displacement (*x*)	*L*^2^
*f*_*B*_	Fraction of nonadsorbed tracers	dimensionless
*f*_*S*_	Fraction of adsorbed tracers	dimensionless
*q*_*A*_	Ratio between adsorption and desorption rates	dimensionless
*q*_*G*_	Geometrical factor of adsorption isotherm	dimensionless
*q*_*T*_	Ratio between adsorption/desorption and bulk diffusivities	dimensionless
*r*_*A*_	Adsorption rate	*T*^–1^
*r*_*D*_	Desorption rate	*T*^–1^
*s*	Specific surface area	*L*^–1^
*t*_*B*_	Bulk (B) region residence time	*T*
*t*_*S*_	Surface (S) region residence time	*T*
⟨*x*^2^⟩	Mean square displacement in the *x* direction	*L*^2^
*A*_*S*_	Area of the surface (S) region	*L*^2^
*C*_*AD*_	Concentration of adsorbed tracers	*L*^–3^
*C*_*NA*_	Concentration of nonadsorbed tracers	*L*^–3^
*D*_*B*_	Bulk diffusion coefficient	*L*^2^*T*^–1^
*D*_*ef*_	Effective diffusion coefficient	*L*^2^*T*^–1^
*F*_*A*_	Area form factor	dimensionless
*F*_*P*_	Perimeter form factor	dimensionless
*V*	Volume of a sample	*L*^3^
*V*_*B*_	Volume of the pore bulk (B) region	*L*^3^
*V*_*P*_	Pore Volume	*L*^3^
*V*_*S*_	Volume of the surface (S) region	*L*^3^
*W*	Pore width	*L*
*W*_*E*_	Effective pore width	*L*
β	Slope of adsorption isotherm	dimensionless
ϵ	Porosity	dimensionless
θ	Surface coverage with tracers	*L*^–2^
τ	Tortuosity	dimensionless
τ_*app*_	Apparent tortuosity factor	dimensionless
τ_*geom*_	Geometric tortuosity factor	dimensionless

## Model and Methods

### Basic features of porous media

**Figure 1:**
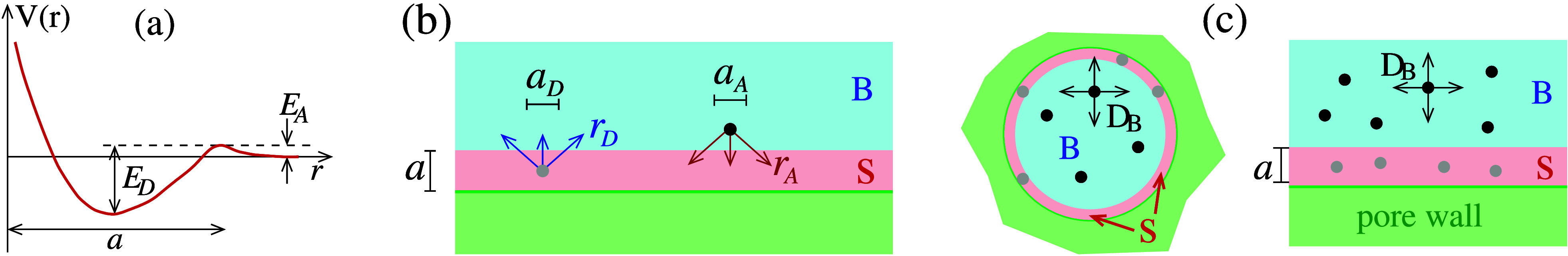
(a) Typical interaction energy as a function of the distance of the tracer from the pore wall. (b) Adsorbed (gray) and nonadsorbed (black) tracers, their transitions (S → B and B → S), and the respective rates. The root-mean-square displacements in the *x* direction of the membrane model are indicated by horizontal bars. (c) Cross and longitudinal sections of a pore with the indicated diffusion coefficient in the bulk.

We consider a material with porosity ϵ and specific surface area *s* (defined as the surface area per unit volume of a sample), whose pores are filled with a fluid where soluble tracers execute molecular diffusion. The concentration is low, so the interactions between the tracers are neglected. However, the tracers interact with the pore walls with the typical one-dimensional potential shown in Figure 1(a). If there are variations in the pore width and in the curvature of the walls throughout the sample, the parameters in the potential of Figure 1(a) may be interpreted as appropriately averaged values.

Each pore has two regions, bulk (B) and surface (S), as shown in Figures 1(b)-(c). The region S, adjacent to the pore walls, has the same width *a* of the range of the tracer-wall interaction [Figure 1(a)], so the tracers in region S are adsorbed. When a tracer is in region B, its interaction with the pore walls is negligible, so it is nonadsorbed.

These definitions allow us to distinguish systems with wide and narrow pores: in the former, the volume *V*_*S*_ of region S is much smaller than the total pore volume *V*_*P*_; in the latter, the tracers interact with the pore walls in a non-negligible fraction of the pore volume, so *V*_*S*_ ∼ *V*_*P*_. In a sample of volume *V*, *V*_*P*_ = *ϵV* and *V*_*S*_ = *asV*, where the interaction range *a* may be an average value, as explained above. Defining the effective pore width as
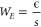
2we have
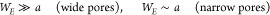
3

Observe that the specific area is usually obtained from gas adsorption, so the proportionality between *V*_*S*_ and *s* is reasonable only if steric hindrance weakly affects the tracer motion. Otherwise, the accessible surface area for the tracer may be very different from the accessible area to small gas molecules.^13,15,16^

### Adsorption and Desorption Transitions

The adsorption rate *r*_*A*_ is the number of tracers that move from B to S per unit surface area per unit time [Figure 1(b)]. Assuming first order adsorption,
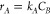
4where *k*_*A*_ is a rate constant and *C*_*B*_ is the tracer concentration (number of tracers per unit volume) in region B.

The desorption rate *r*_*D*_ is the number of tracers that move from S to B per unit surface area per unit time. First order desorption implies that this rate is proportional to the concentration *C*_*S*_ defined as the number of adsorbed tracers per unit volume of region S:
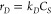
5where *k*_*D*_ is another rate constant. Alternatively, we could write *r*_*D*_ = *k*_*D*_^′^θ, where the surface coverage θ is the number of adsorbed tracers per unit area of S and *k*_*D*_^′^ is another rate constant (*C*_*S*_ = θ/*a*, *k*_*D*_ = *ak*_*D*_^′^).

The adsorption ratio *q*_*A*_ is defined as the ratio between the rate constants of adsorption and desorption. If those rates have temperature activated forms with the same prefactor, we obtain
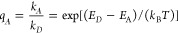
6where *E*_*A*_ and *E*_*D*_ are activation energies [Figure 1(a)], *k*_B_ is the Boltzmann constant and *T* is the temperature. In general we expect that the desorption rate is smaller than that of adsorption, so that *q*_*A*_ > 1. If the thermal energy is low compared to *E*_*D*_ – *E*_*A*_, then *q*_*A*_ ≫ 1.

The adsorption isotherm may be determined in terms of the above rates and of the parameters *W*_*E*_ and *a*, independently of particular features of the porous medium, such as tortuous paths and interconnections. A detailed specification of the medium geometry is necessary only to calculate the effective diffusion coefficient.

### Scheme of a Porous Membrane

**Figure 2:**
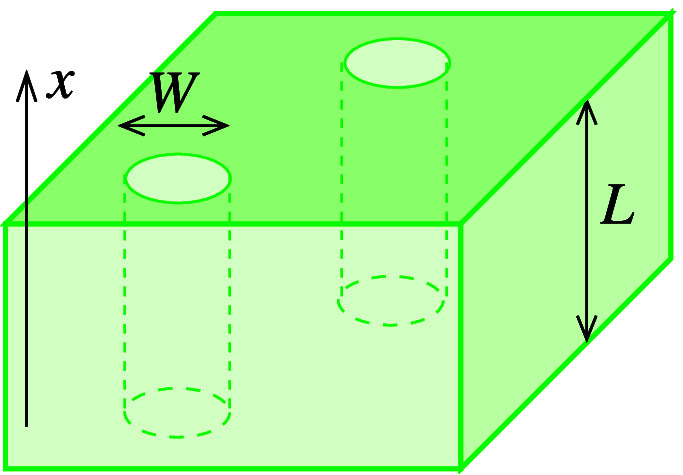
Scheme of a simple porous membrane.

For the calculation of the effective diffusion coefficient, a simple model of a porous membrane is proposed in Figure 2, with a bundle of nonintersecting parallel pores with characteristic width *W* and length *L* ≫ *W*. The direction perpendicular to the membrane is the *x* direction, along which the MSD is calculated. In this model, there is no geometrical tortuosity.

The porosity ϵ and the specific surface area *s* can be respectively written as ϵ = *F*_*A*_*W*^2^ and *s* = *F*_*P*_*W*, where *F*_*A*_ and *F*_*P*_ are form factors of order 1 (e.g., *F*_*A*_ = π/4 and *F*_*P*_ = π for a circle). A simple algebra shows that
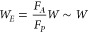
7

In pores with circular and square shapes, . When the pore cross section has a small aspect ratio, the ratio of form factors in Eq. 7 usually is sligthly smaller, . This is the ratio between the effective width *W*_*E*_ and the actual pore width *W*. A failure of this interpretation is expected only if the aspect ratio of the pore is large (e.g., a fracture) or if the surface area *s* measured by gas adsorption is significantly different from the area accessible to the tracers (significant hindrance effects^13,15,16^).

Our model will be applied to diffusion of different molecules in mesoporous silica samples in which average pore diameters and average neck diameters were measured.^29,30^ The ratio between *W*_*E*_ and the average neck diameters is in the range 0.27–0.32, whereas the ratio between *W*_*E*_ and the average pore diameters is 0.14–0.27. These values support the extension of our interpretation of *W*_*E*_ to media with tortuous geometries and with pore intersections.

### Tracer Diffusion

The tracer moves in region B with diffusion coefficient *D*_*B*_, which is assumed to have the same value as in a nonconfined fluid; see Figure 1(c). Diffusion of adsorbed tracers (in region S) is neglected in this model. However, here we assume that adsorption and desorption transitions may also contribute to the MSD in the pore length direction, as explained below.

In molecular diffusion processes, the tracers frequently collide with fluid molecules that fill the pore space, so tracer diffusion represents the random exchange of their positions in all spatial directions. This exchange also occurs in adsorption and desorption transitions, so that the tracer displacement is not expected to be restricted to the direction perpendicular to the pore wall. Thus, tracer displacements may have components along the directions of the pore walls and, consequently, contribute to the MSD, as shown in Figure 1(b). We then define:

*a*_*A*_^2^: MSD along the *x* direction in an adsorption transition;

*a*_*D*_^2^: MSD along the *x* direction in an desorption transition.

The values of *a*_*A*_ and *a*_*D*_ depend on how the positions of the tracer and of the fluid molecules are exchanged. This is a complex problem related to the details of the tracer-fluid interactions and to the organization of fluid molecules near the pore walls; for instance, water molecules near pore walls may have low or negligible mobility and an organization different from that in the bulk.^26,35,36^ However, since the range of tracer-wall interaction is *a* [Figure 1(a)], we expect *a*_*A*_ ∼ *a* and *a*_*D*_ ∼ *a*.

Furthermore, we assume that there is no correlation between the displacements in adsorption and desorption processes, as well as no correlation in the displacements of lengths ∼ *a* in the bulk that occur before adsorption or after desorption. In other words, no memory effect is present after displacements of order *a* or larger. Diffusion models that account for such memory effects may have anomalous diffusion.^24,25^

The contributions of adsorption and desorption events to the MSD in our model have a parallel with those of Knudsen diffusion. In the latter, the interactions between gas molecules are negligible, but their velocities change during the collisions with the pore walls. Diffuse scattering of a tracer is generally assumed, in which the angle of reflection (after tracer-wall collision) is uncorrelated with the angle of incidence because the adsorption times are long enough for the energy to be redistributed among the degrees of freedom.^4^ This parallels our assumption of uncorrelated displacements in adsorption, in desorption, and in the bulk. The main difference is the order of magnitude of the contributions to the MSD: in Knudsen diffusion, the uncorrelated displacements are of the order of magnitude of the pore width *W*; in our molecular diffusion model, the displacements are of order *a* because the transitions represent exchanges between positions where the tracer interacts and does not interact with the pore walls.

### Methods of Solution and Quantities of Interest

A scaling approach is used to calculate the effective diffusion coefficient. The method is similar to that of a recent work on a random walk model in the pores of a packing of spheres.^37^ This type of phenomenological approach facilitates the interpretation of different diffusion regimes^38^ without solving diffusion equations (although those equations might be written from the model rules). We focus on the diffusion across the sample in a given *x* direction, where the MSD ⟨(Δ*x*)^2^⟩ is calculated. The effective diffusion coefficient in the steady state is given as
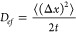
8

The MSD is usually written in terms of bulk and surface diffusion coefficients,^37,39−41^ while the adsorption–desorption transitions are assumed to be responsible only for setting the equilibrium concentrations. However, we will show that this is a reasonable approximation only for wide pores, so here the MSD is written with contributions of bulk diffusion and of adsorption and desorption transitions:
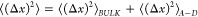
9The summation of these contributions is possible because the displacements of length ≳ *a* in the bulk, in the adsorption, and in the desorption processes are uncorrelated.

Once the MSD is determined in the steady state, we can calculate the effective diffusion coefficient [Eq. 8] and, from Eq. 1, the apparent tortuosity due to adsorption.

## Results and Discussion

### Tracer Partitioning between Bulk and Surface

The concentrations of adsorbed and nonadsorbed tracers, respectively denoted as *C*_*AD*_ and *C*_*NA*_, are the numbers of tracers per unit pore volume (or unit volume of the solution), i.e. they are normalized by the total pore volume *V*_*P*_. They differ from the concentrations *C*_*B*_ and *C*_*S*_ [Eqs. 4 and 5], which were normalized by the volumes of regions B and S (consistently with the usual relations for first order processes), but they are related as *V*_*B*_*C*_*B*_ = *V*_*P*_*C*_*NA*_ and *V*_*S*_*C*_*S*_ = *V*_*P*_*C*_*AD*_.

In a sample with volume *V*, we recall that *V*_*P*_ = *ϵV* and *V*_*S*_ = *asV*. Hence, the rates in Eqs. 4 and 5 can be written as  and , respectively. In equilibrium, the adsorption and desorption rates are equal (*r*_*A*_ = *r*_*D*_), which leads to the (Henry) adsorption isotherm
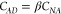
10where
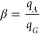
11is the dimensionless slope given by the ratio between the adsorption ratio *q*_*A*_ [Eq. 6] and the geometrical factor
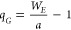
12where Eq. 2 was used. Using Eq. 6, Eq. 10 may be written in terms of activation energies and temperature.

The parameter β in Eq. 10 is the ratio of the loadings of pore surface and pore bulk. The derivation of that isotherm is widely known, but the important point here is the split of β into two dimensionless factors, one of them dependent on the rates of chemical processes and the other related to the pore and tracer geometries. The geometrical factor *q*_*G*_ is the ratio of the volumes of regions B and S. In wide pores, Eq. 3 implies *q*_*G*_ ≈ *W*_*E*_/*a* ≫ 1; in narrow pores, *q*_*G*_ ≲ 1.

As explained above, the definition of the effective pore width [Eq. 2] is also reasonable when there is a dispersion in the pore widths and when the pores are tortuous, so the prediction of the isotherm slope from Eqs. 6, 11, and 12 is expected to be a reasonable approximation. However, a failure may occur if the accessible surface area for the tracers is much smaller than that accessible for the gas used to measure the specific area *s* (i.e., hindrance effects).

The fractions of the time in which a single tracer is located in regions B and S are denoted as *f*_*B*_ and *f*_*S*_, respectively. They are proportional to the corresponding concentrations *C*_*NA*_ and *C*_*AD*_ in the steady state. Eq. 10 gives *f*_*S*_ = *βf*_*B*_ and, since *f*_*B*_ + *f*_*S*_ = 1, we obtain
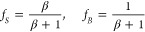
13

This allows the identification of two regimes of tracer residence in which β is a scaling variable: for β ≪ 1, there is dominant bulk residence of the tracers; for β ≫ 1, there is dominant surface residence, but with dominant bulk displacement because surface diffusion is neglected. In the latter case, the waiting times of the tracer in the pore walls are long compared to the times of motion in the pore bulk; a typical case is strong adsorption, in which *q*_*A*_ is sufficiently large so that *q*_*A*_/*q*_*G*_ is large even in wide pores.

### Scaling of the MSD

We determine the MSD by separately calculating each contribution in Eq. 9. This is achieved by using the residence times of the tracer in each region after a diffusion time *t*, *t*_*S*_ = *f*_*S*_*t* in S and *t*_*B*_ = *f*_*B*_*t* in B.

The first contribution accounts for the displacements between points of the pore bulk:
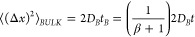
14

The bulk MSD is zero in the case of no desorption (*k*_*D*_ = 0, β → *∞*) because all tracers will eventually reach the surface and remain immobile in the steady state.

In order to determine the second contribution, we need the number of adsorption transitions in the time *t*. In a sample of volume *V*, using Eq. 4, that number is *r*_*A*_*A*_*S*_*t*, where *A*_*S*_ = *sV* is the area of region S. The number of desorption transitions in the same volume and in the same time has the same value. Each transition contributes with *a*_*A*_^2^ and *a*_*D*_^2^ to the MSD in the *x* direction, respectively. Summing these contributions and dividing by the total number of tracers in the sample volume, (*C*_*NA*_+*C*_*AD*_)*V*_*P*_, with *V*_*P*_ = *ϵV*, we obtain
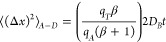
15where we define
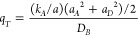
16

The factor *k*_*A*_/*a* may be interpreted as an adsorption frequency, whereas *D*_*B*_/*a*^2^ is a diffusion frequency in the bulk. For *a*_*A*_ ∼ *a*_*D*_ ∼ *a*, the factor *q*_*T*_ in Eq. 16 is the ratio between these frequencies. If the adsorption barrier is negligible and we consider that tracer motion takes place by exchange of positions with fluid molecules, the tracers adjacent to region S are expected to be adsorbed with a frequency similar to that of their diffusion in the bulk; in this case, we expect *q*_*T*_ ∼ 1. Otherwise, for high adsorption barriers (*E*_*A*_ ≫ *k*_B_*T*), we expect *q*_*T*_ ≪ 1, i.e. a displacement of the tracer to region S is much less frequent than the same displacement in the bulk.

The relative role of the adsorption and desorption transitions is given by the ratio
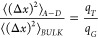
17

In wide pores (*q*_*G*_ ≫ 1) or with high adsorption barriers (*q*_*T*_ ≪ 1), the contribution of those transitions for the MSD is negligible. Instead, in narrow pores (*q*_*G*_ ≲ 1) and with small adsorption barriers (*q*_*T*_ ∼ 1), those transitions give a contribution to the MSD of the same order of that of bulk displacement. This result does not depend on the energy barrier of desorption.

Since the adsorption requires exchanges of positions of the tracer and of the fluid molecules, the corresponding energy barrier depends on the interactions of both chemical species. This means that, for the adsorption to occur, the displacement of fluid molecules near the pore wall is necessary. If the fluid molecules are strongly bound to the pore walls, that energy barrier is probably high; this is expected to occur, for instance, with the almost immobile water monolayers in the walls of silica nanopores.^35^ In such cases, the contribution of adsorption–desorption transitions to the MSD is expected to be small even in narrow pores because *q*_*T*_ ≪ 1. Otherwise, if the interaction between the fluid molecules and the pore walls is weak, the condition *q*_*T*_ ∼ 1 may be satisfied.

With the expressions obtained for the two contributions, we can write the MSD as
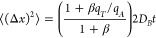
18

### Apparent Tortuosity

The effective diffusion coefficient through the membrane is given as *D*_*ef*_=ϵ⟨(Δ*x*)^2^⟩/(2*t*), where the porosity ϵ accounts for the constraint that the tracer moves only in the pore volume (but still without effects of the geometrical tortuosity). Using the MSD in Eq. 18 and Eq. 1, the adsorption is responsible for an apparent tortuosity factor
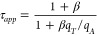
19

Whenever *βq*_*T*_/*q*_*A*_ ≪ 1, the apparent tortuosity has the standard form
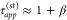
20i.e. it can be predicted by the slope of the adsorption isotherm, as shown in previous works.^27,28^ In order to determine the limits of applicability of this relation and possible extensions, we begin recalling that *q*_*T*_ ≲ 1 and, in general, *q*_*G*_ ≳ 1 (except in extremely narrow pores, where *q*_*G*_ may be much smaller than 1). The apparent tortuosity is then separately analyzed in wide and narrow pores.

#### Wide Pores (*q*_*G*_ ≫ 1)

Eq. 11 implies β ≪ *q*_*A*_, so the standard relation in Eq. 20 is valid. However, the approximation *q*_*G*_ ≈ *W*_*E*_/*a* gives a more general form:
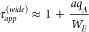
21

The second term in the right-hand side is the slope β, but Eq. 21 allows to estimate this quantity from microscopic parameters without measuring the isotherm.

In the case of dominant bulk residence (β ≪ 1), τ_*app*_^(*wide*)^ ≈ 1, i.e. there is no apparent tortuosity. If any tortuosity is measured in a porous medium in this regime, that is a consequence only of the pore geometry. Otherwise, for dominant surface residence (β ≫ 1), τ_*app*_^(*wide*)^ ≫ 1, which is a consequence of the long delay in the motion when the tracers are adsorbed. This was the case of MgCl_2_ diffusion in the cellulosic fibers studied by Tozzi et al.^28^

#### Narrow Pores (*q*_*G*_ ∼ 1)

Eq. 11 implies that β is of the same order as *q*_*A*_, so an approximation for the apparent tortuosity is
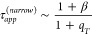
22

For high adsorption barriers, *q*_*T*_ ≪ 1, we obtain the standard relation in Eq. 20. However, here β has to be determined directly from the adsorption isotherm (measured or calculated); the wide pore approximation in Eq. 21 cannot be used. For low adsorption barriers, *q*_*T*_ ∼ 1, the standard relation overestimates τ_*app*_ because it neglects the contributions of adsorption and desorption transitions to the MSD; see Eq. 15. Thus, all terms in Eq. 19 must be considered or, possibly, the approximation in Eq. 22. The order of magnitude of the apparent tortuosity may be predicted by Eq. 20, but this will be only a rough approximation.

Finally, observe that the rates of adsorption and desorption are expected to have thermally activated forms, so the parameters *q*_*A*_ and *q*_*T*_ in Eq. 19 have such forms. Thus, in all the scaling regimes where τ_*app*_ > 1, the apparent tortuosity depends on the operation conditions (e.g., temperature). When a material has a geometrical tortuosity, an additional reduction of the effective diffusion coefficient is expected and the tortuosity factor τ obtained from Eq. 1 is written as
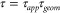
23

Some of the above approximations for τ_*app*_ are helpful for specific systems.

### Applications to Diffusion in Mesoporous Silica

#### Summary of Recent Experimental and Theoretical Results

Casillas et al.^29^ recently reported results on diffusion of alkaline chlorides and water in silica samples with nominal pore diameters ranging from 3 to 30 nm. First, they obtained morphological quantities such as porosity, specific surface area, and average pore sizes from adsorption techniques, which are shown in Table 2; the specific area values in nm^–1^ were obtained from the reported values in m^2^/g, the specific volumes in cm^3^/g, and the porosities. From ϵ and *s*, we calculated the effective pore size *W*_*E*_ [Eq. 2]. Diffusion coefficients were obtained in several values of the solution pH by conductivity measurements. In order to analyze data in similar chemical conditions for all five samples studied by Casillas et al.,^29^ we consider the coefficients of LiCl diffusion in pH = 5.2 obtained at short experimental times. In Table 3, we present the ratios between those coefficients in the mesoporous silica and in the bulk liquids with the reported uncertainties.

**Table 2:** Morphological Quantities of Mesoporous Silica Samples from Adsorption Techniques

Sample	Nominal pore diameter (nm)^42^	Average pore diameter (nm)^29^	Average neck diameter (nm)^29^	Porosity ϵ^29^	Specific surface area *s*(nm^–1^)^29^	Effective pore width *W*_*E*_ (nm)
Q3	3	2	2	0.38	0.71	0.54
Q6	6	8.0	5.5	0.57	0.36	1.6
Q10	10	20.4	10.6	0.68	0.20	3.4
Q15	15	38.3	18.8	0.67	0.13	5.2
Q30	30	57.7	31.3	0.66	0.069	9.6

**Table 3:** Ratios between Diffusion Coefficients in the Mesoporous Silica and in the Bulk Liquids, and Fractions of Adsorbed Alkanes

Sample	*D*_*eff*_/*D*_*B*_ (LiCl, pH = 5.2)^29^	*D*_*B*_/*D*_*eff*_ (alkanes)^30^	*f*_*S*_ (alkanes)^43^
Q3	0.0102 ± 0.0008	29.4	0.35
Q6	0.28 ± 0.02	3.1	0.20
Q10	0.45 ± 0.03		
Q15	0.55 ± 0.08	1.65	<0.1
Q30	0.63 ± 0.08	1.44	<0.1

In the sample with the narrowest pores, Q3, the same average pore size was estimated from adsorption and desorption isotherms. In the other samples, the different adsorption and desorption curves respectively led to estimates of average pore diameters (adsorption) and average neck diameters (desorption), as shown in Table 2. The neck diameters of all samples are much larger than the effective diameter of 0.152 nm of Li^+^ ions,^44^ which suggests that the bottlenecks of the pore network are accessible to those ions. The ratios between *W*_*E*_ and the neck diameters are in the range [0.27,0.32], whereas the ratios between *W*_*E*_ and the average pore diameters are in the range [0.14,0.27]. They are consistent with pore shapes whose aspect ratios are near 1 and where pore intersections have small effects. These observations are valid even for Q3, in which both ratios are 0.27, i.e. very close to the value 0.25 of straight and aligned pores of circular or square shape. Thus, we understand that our model can be a reasonable approximation to distinguish geometrical and adsorption effects on the diffusion in these media.

Working with the same mesoporous silica samples (except Q10), Linck et al.^30^ recently obtained self-diffusion coefficients of some organic molecules using ^1^H NMR. For the majority of those molecules (acetone, cyclohexane, *n*-pentane, *n*-heptane, 2-propanol), the measured ratios *D*_*eff*_/*D*_*B*_ were approximately the same in each sample. Those values are presented in Table 3. The uncertainties were not listed by Linck et al.,^30^ but inspection of the plots in their Supporting Information indicate that they are in the range 5%–10%.

Alkane self-diffusion is considered as a suitable process to probe the geometrical tortuosity of porous materials commonly used in heterogeneous catalysis because those molecules weakly interact with the solid walls. For instance, using nuclear magnetic resonance (NMR) methods, D’Agostino et al.^45^ studied self-diffusion of cyclohexane, *n*-hexane, *n*-octane, and *n*-decane in samples of TiO_2_, γ–Al_2_O_3_, and SiO_2_ with average pore sizes 13–22 nm. In each of those materials, they obtained tortuosity values that varied less than 2% among the four alkanes. However, compared to their work, the mesoporous silica samples Q3 and Q6 have narrower pores.

Recent ab initio calculations of the interaction energy of alkanes and silica mesopores predict significant retention of those molecules in the pore walls of the samples with the narrowest pores.^43^ For instance, in sample Q3, more than 30% of the *n*-alkanes and more than 40% of the cycloalkanes are expected to be adsorbed. In sample Q6, both fractions decrease to approximately 20%, and they are smaller than 10% in the samples with the widest pores (Q15 and Q30). The average fractions of adsorbed *n*-alkanes reported by Chevallier-Boutell et al.^43^ are estimates of the fraction *f*_*S*_ in our modeling [Eq. 13]; see Table 3.

#### Application of the Model to Alkane Self-Diffusion

In the silica samples Q15 and Q30, the predicted small adsorbed fractions of *n*-alkanes suggest that their self-diffusion actually gives the geometrical tortuosities. In the samples Q3 and Q6, the fractions of adsorbed alkanes in Table 3 are substituted in Eq. 13 and give β = 0.54 and 0.25, respectively. In the other samples, β = 0 is assumed, i.e. negligible adsorption. Considering that the pore walls are hydrofilic, we assume that there is an energy barrier for adsorption that leads to *q*_*T*_ ≪ 1. This allows the use of the standard relation of Eq. 20, in which the apparent tortuosity depends only on β and which is valid for narrow or wide pores. The (total) tortuosity factors τ are obtained from Eq. 1, the measured values of porosity (Table 2), and the effective diffusion coefficients (Table 3). The geometrical tortuosity is then calculated from Eq. 23.

**Figure 3:**
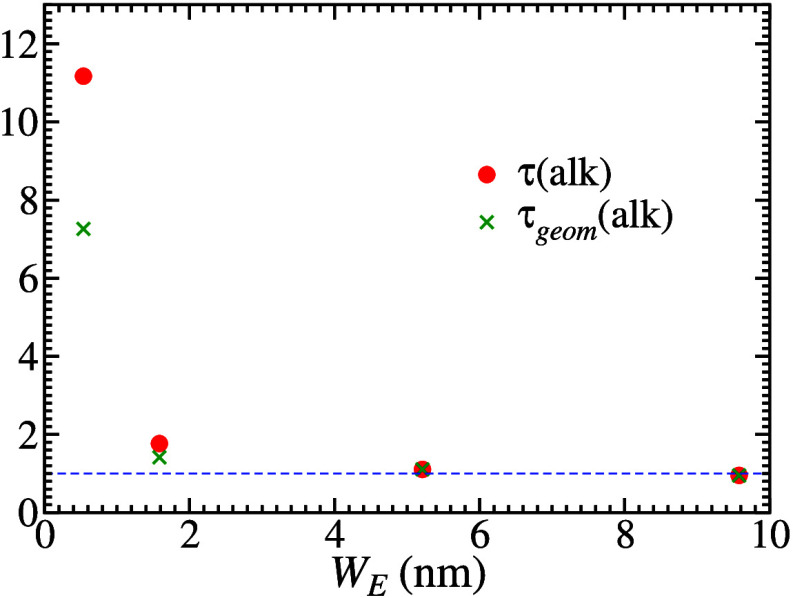
Total tortuosity (green crosses) and geometrical tortuosity (red circles) of mesoporous silica samples obtained after corrections of alkane diffusion data in the samples with the smallest pores (*W*_*E*_ < 2 nm). The blue dashed line marks the value 1 of both quantities.

Figure 3 show τ and τ_*geom*_ of the mesoporous silica samples obtained from this treatment of the alkane diffusion data, as a function of the effective pore width (Table 2). The geometrical tortuosities of samples Q15 and Q30 are negligible, i.e. τ_*geom*_ ≈ 1. In Q6, τ_*app*_ = 1.25 indicates a weak (but non-negligible) effect of the adsorption and τ_*geom*_ ≈ 1.4 suggests that the sample has a slightly tortuous geometry. However, in Q3, τ_*geom*_ ≈ 7.3 and τ_*app*_ = 1.54 indicate that the tortuous geometry is the main reason for the small diffusion coefficient in that sample, with the adsorption playing a less important role.

#### Wide Pore Approximation Applied to LiCl Diffusion

Our model focus on the diffusion of Li^+^ ions in the aqueous solutions inside the pores of the silica samples. In this molecular diffusion process, the adsorption of an ion in a pore wall is expected to be accompanied by displacement of water molecules from that wall. However, NMR studies show strong interactions between water molecules and the pore walls of mesoporous silica^46^ and molecular dynamics simulations show that water is structured and nearly immobile at the walls of silica nanopores.^35^ Thus, we expect that there is a non-negligible energy barrier for the displacement of those water molecules when the Li^+^ ions are adsorbed. This implies *q*_*T*_ ≪ 1, as previous explained. Moreover, no estimate of the isotherm slope β or of the fraction *f*_*S*_ of adsorbed ions is known in this case, so we have to use the relations with the adsorption ratio and with the medium geometry developed in this work.

Our first step is to obtain bounds of the model parameters from the data of the sample Q30, which has the widest pores. Using the diffusion coefficients and the porosity in Tables 2 and 3, Eq. 1 gives
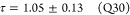
24

Since the pores are wide, Eq. 21 and the estimate of *W*_*E*_ in Table 2 give
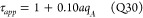
25with *a* in nanometers. From these relations we can calculate bounds for the factor *aq*_*A*_:

(a) Eq. 24 gives a maximal possible tortuosity factor τ = 1.18, while the minimal possible geometrical tortuosity factor is τ_*geom*_ = 1. Thus, Eq. 23 gives τ_*app*_ ≤ 1.18 and Eq. 25 gives *aq*_*A*_ ≤ 1.8 nm.

(b) The minimal possible value of the adsorption ratio is *q*_*A*_ = 1, which means absence of an adsorption well. The minimum thickness *a* of region S is expected to be of the order of the diameter of a Li^+^ ion. Considering the effective radius of that ion,^44^ we obtain *a* ≥ 0.15 nm, which gives *aq*_*A*_ ≥ 0.15 nm.

These two limiting cases then give
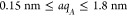
26

**Figure 4:**
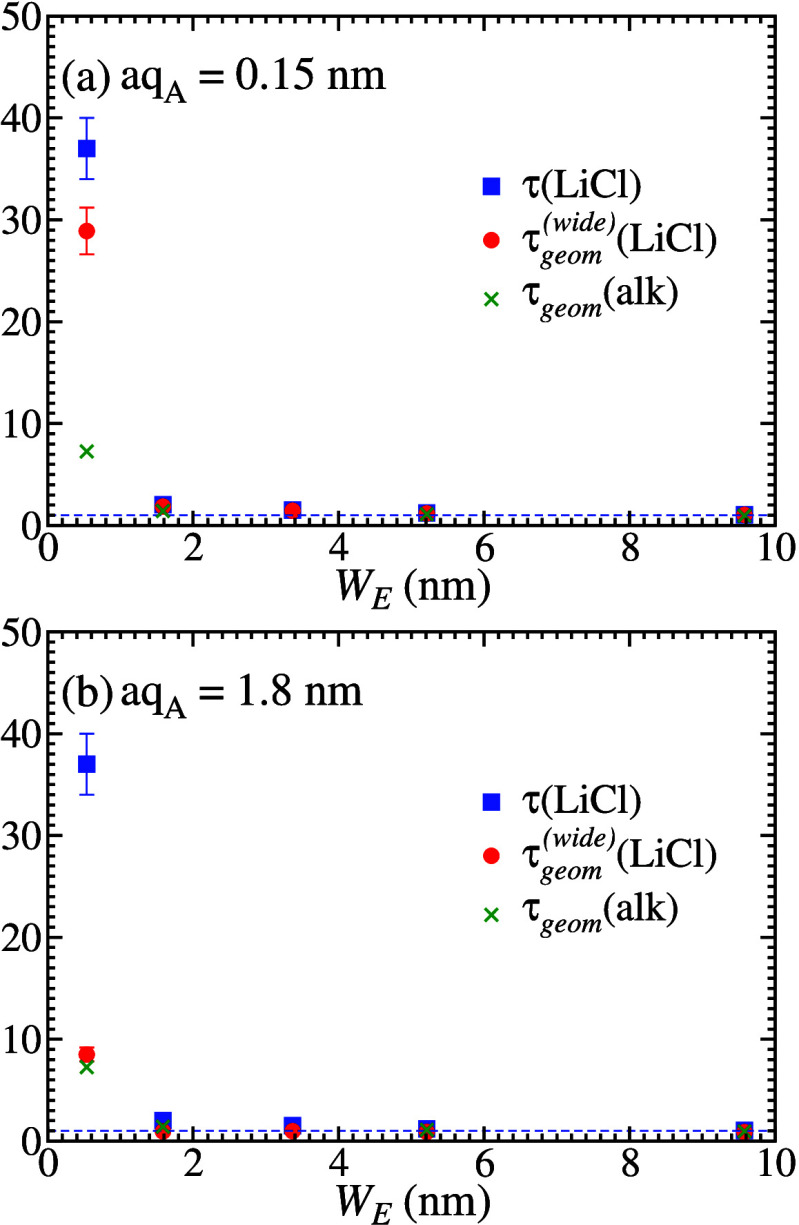
Total tortuosity from LiCl diffusion (blue filled squares), geometrical tortuosity from LiCl diffusion in the wide pore approximation (red filled circles), and geometrical tortuosity from alkane diffusion (green crosses) as functions of the effective width of mesoporous silica. The indicated values of *aq*_*A*_ were used to calculate the geometrical tortuosity from LiCl diffusion data. The blue dashed line marks the value 1 of both quantities.

Now we assume that the tracer-wall interaction does not change in the other silica samples, which implies that the bounds of *aq*_*A*_ in Eq. 26 remain the same. As a first approximation, for all samples we use the wide pore formula for the apparent tortuosity [Eq. 21], the bounds for *aq*_*A*_, and the values of *W*_*E*_ in Table 2. From Eq. 1 and the data in Tables 2 (porosity) and 3 (diffusion coefficients), we obtain the experimental values of τ. Eq. 23 is then used to calculate the geometrical tortuosities, which are denoted as τ_*geom*_^(*wide*)^ to highlight the application of the wide pore approximation. The values of τ and τ_*geom*_^(*wide*)^ are shown in Figures 4(a) and 4(b) for all samples, respectively using *aq*_*A*_ = 0.15 nm and *aq*_*A*_ = 1.8 nm [Eq. 26]. For comparison, the geometrical tortuosities obtained from alkane diffusion after the corrections for adsorption are also shown (the same data as in Figure 3).

In Figure 4(a), the LiCl and alkane values are nearly the same in most samples, but there are very large deviations in the estimates for Q3. This suggests that the lower bound of Eq. 26 does not provide a suitable correction for the adsorption of Li^+^ ions. Observe that this lower bound is obtained with an adsorption ratio *q*_*A*_ = 1, which is the unlikely case of no adsorption well. Instead, in Figure 4(b), the upper bound of Eq. 26 leads to small deviations in the geometrical tortuosities from LiCl and alkane diffusion. In sample Q3, τ_*app*_ ∼ 4 indicates significant adsorption effects, contrary to the case of the alkanes. The only concern about the present comparison is that the wide pore approximation is being applied.

#### Extended Model of LiCl Diffusion

**Figure 5:**
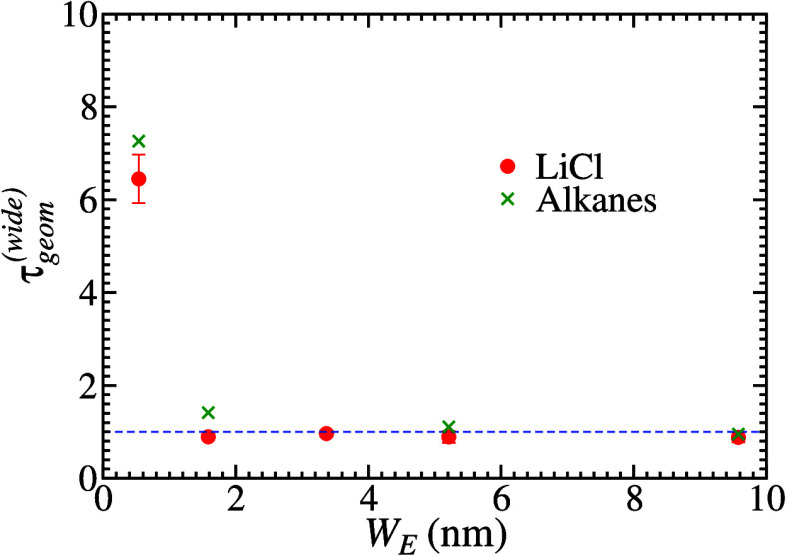
Geometrical tortuosities of mesoporous silica samples obtained after corrections of LiCl diffusion data (red circles) and alkane diffusion data (green crosses). The blue dashed line marks the value 1 of both quantities.

In order to treat the adsorption effects in LiCl diffusion without the wide pore approximation, additional assumptions are necessary: (i) motivated by the former results, the upper bound *aq*_*A*_ = 1.8 nm of Eq. 26 is considered; (ii) the range of the tracer-wall interaction is roughly the diameter of Li^+^ ions, *a* ≈ 0.15 nm. These assumptions lead to *q*_*A*_ ≈ 12, so that the parameter β can be determined from Eqs. 11 and (12) without further approximations. Since *q*_*T*_ ≪ 1, the standard relation (20) for the apparent tortuosity is valid and the geometrical tortuosities can be obtained from Eq. 23. They are shown in Figure 5 and, for comparison, the values obtained from alkane diffusion after the corrections for adsorption are also shown.

Here we also obtain reasonable agreement with the predictions from alkane diffusion in samples Q3, Q15, and Q3, the two latter with very small τ_*geom*_. In sample Q6, the treatment of LiCl diffusion data suggests τ_*geom*_ slightly smaller than that of treated alkane data, but both are representative of pore networks with low (possibly negligible) tortuosities. Also recall that the alkane diffusion data have uncertainties in the range 5%–10%.

Again we observe that sample Q3 has a significant geometrical tortuosity compared to the other samples. However, the values of τ_*geom*_ in Figure 5 are significantly smaller than the values of τ directly obtained from LiCl and alkane diffusion data, shown in Figures 3, 4(a), and 4(b). This shows the importance of the treatment of the values of τ to exclude the effects of adsorption. For the Li^+^ ions, τ_*app*_ ∼ 5 confirms that the adsorption effect is much more important than that for alkanes, where τ_*app*_ ≈ 1.5.

The above approximations also permit to estimate the difference of activation energies in the adsorption of Li^+^ ions. Using Eq. 4 with *q*_*A*_ ≈ 12 and *T* = 298.15 K,^29^ we obtain *E*_*D*_ – *E*_*A*_ ∼ 0.06 eV. This value may eventually be checked with ab initio methods.

### Relations with Other Works

Some laboratory studies have also shown that apparent tortuosity factors might be used to quantify the delay in the diffusion caused by adsorption. Examples are studies of diffusion of organic molecules in xerogel,^47^ manganese chloride in cellulosic fibers,^28^ and dissolved micropollutants (phthalic acid, bisphenol A, and others) in pores of activated carbon.^23^

These observations motivated the proposal of models for the apparent tortuosity. The simplest models assume that adsorbed tracers are immobile and that the volume in which they are adsorbed is much smaller than the total pore volume (wide pore approximation).^27,28^ Thus, they obtain the apparent tortuosity of Eq. 20, which depends solely on the slope β of the adsorption isotherm. These models were recently used in studies of gas diffusion in kerogen.^10,48^ Alternative approaches were also developed to distinguish the effects of adsorption and of the geometric tortuosity in studies of gas or liquid diffusion.^26,49^

We understand that there are two important differences between those approaches and the present work. First, the dependence of the initial slope of the adsorption isotherm (β in our notation) was determined here in terms of physicochemical and geometrical parameters. This was essential for our applications to diffusion in mesoporous silica. Second, we showed that apparent tortuosities of samples with narrow pores may be affected by contributions of frequent adsorption–desorption transitions to the MSD. We had reasons to neglect these contributions in our applications, but it is important to anticipate that they may play a role in systems with narrow pores.

On the other hand, the contribution of adsorption and desorption to the MSD predicted here for a molecular diffusion process has a parallel with Knudsen diffusion of gas molecules, in which diffuse scattering is generally assumed for the collisions with the pore walls.^4^ The difference is quantitative, since here the displacements of length ∼ *a* are uncorrelated, while the uncorrelated displacements in Knudsen diffusion are of the order of the pore width.

Finally, there are some problems of diffusion in porous materials in which extensions of our approach would be necessary. For instance, diffusion of adsorbed species may contribute to the MSD and reduce the delay caused by the adsorption, as shown in experiments and modeling approaches.^12,40,50−52^ More complex situations are those in which the pores have many interconnections and broad size distributions, where other approaches may be necessary to determine the tortuosity factor.^22,53^

## Conclusion

We introduced a model of tracer diffusion and adsorption in a simple model of a porous membrane. The delay in the tracer displacement due to adsorption is quantified by an apparent tortuosity τ_*app*_. A scaling approach is used to determine the ratio of surface and bulk loadings (adsorption isotherm), to separate the contributions of pore bulk diffusion and adsorption–desorption transitions to the mean square displacement (MSD), and finally to obtain an expression for τ_*app*_.

In the expression of the adsorption isotherm, we highlight the presence of factors related to adsorption–desorption transitions and to the pore geometry, which helps to discuss results for wide and narrow pores. In narrow pores and with low energy barriers for adsorption, we show that adsorption–desorption transitions and bulk diffusion give contributions of the same order to the MSD. The general expression of the apparent tortuosity then depends on details of the microscopic processes taking place near the pore walls. However, in wide pores and in cases of high adsorption barriers, the simple relation τ_*app*_ = 1 + β is recovered, where β is the ratio of surface and bulk loadings, in agreement with previous works. In such cases, the advance of our treatment is to split this parameter into a geometrical factor and a ratio of adsorption and desorption rates.

The geometrical tortuosities of mesoporous silica samples with nominal pore diameters 3–30 nm were estimated by applying the model to results of LiCl and alkane diffusion in those samples. The treatment of alkane diffusion data in narrow pores accounts for the adsorbed fractions obtained in recent ab initio calculations. LiCl diffusion data was initially treated by the model in the wide pore approximation and the comparison with the geometrical tortuosities obtained from alkane diffusion helps to find the most suitable parameters for the interaction between Li^+^ ions and the pore walls. The extension of that treatment led to good agreement between the geometrical tortuosities estimated from diffusion and adsorption of very different chemical species. We also obtained a rough estimate of the difference of activation energies of adsorption and desorption of LiCl, which may eventually be tested with ab initio calculations.

## References

[ref1] Adler P. M. Porous media: geometry and transports; Butterworth-Heinemann: Stoneham, MA, USA, 1992.

[ref2] Cussler E. L. Diffusion: mass transfer in fluid systems, 3rd ed.; Cambridge University Press: Cambridge, UK, 2007.

[ref3] Grathwohl P. Diffusion in natural porous media: contaminant transport, sorption/desorption and dissolution kinetics; Springer: New York, USA, 1998.

[ref4] Bukowski B. C.; Keil F. J.; Ravikovitch P. I.; Sastre G.; Snurr R. Q.; Coppens M. Connecting theory and simulation with experiment for the study of diffusion in nanoporous solids. Adsorption 2021, 27, 683–760. 10.1007/s10450-021-00314-y.

[ref5] Coasne B. Multiscale adsorption and transport in hierarchical porous materials. New J. Chem. 2016, 40, 4078–4094. 10.1039/C5NJ03194J.

[ref6] Mueller R.; Zhang S.; Klink M.; Bäumer M.; Vasenkov S. The origin of a large apparent tortuosity factor for the Knudsen diffusion inside monoliths of a samaria-alumina aerogel catalyst: A diffusion NMR study. Phys. Chem. Chem. Phys. 2015, 17, 27481–27487. 10.1039/C5CP04609B. 26426141

[ref7] Wang R.; Bukowski B. C.; Duan J.; Sui J.; Snurr R. Q.; Hupp J. T. Art of architecture: Efficient transport through solvent-filled metal-organic frameworks regulated by topology. Chem. Mater. 2021, 33, 6832–6840. 10.1021/acs.chemmater.1c01536.

[ref8] Wu H.; Schwartz D. K. Nanoparticle tracking to probe transport in porous media. Acc. Chem. Res. 2020, 53, 2130–2139. 10.1021/acs.accounts.0c00408. 32870643

[ref9] Horseman T.; Yin Y.; Christie K. S. S.; Wang Z.; Tong T.; Lin S. Wetting, scaling, and fouling in membrane distillation: State-of-the-art insights on fundamental mechanisms and mitigation strategies. ACS EST Engg 2021, 1, 117–140. 10.1021/acsestengg.0c00025.

[ref10] Afagwu C.; Al-Afnan S.; Patil S.; Aljaberi J.; Mahmoud M. A.; Li J. The impact of pore structure and adsorption behavior on kerogen tortuosity. Fuel 2021, 303, 121261 10.1016/j.fuel.2021.121261.

[ref11] Yang Y.; Wang M. Cation diffusion in compacted clay: A pore-scale view. Environ. Sci. Technol. 2019, 53, 1976–1984. 10.1021/acs.est.8b05755. 30652850

[ref12] Schaefer C. E.; Drennan D.; Nickerson A.; Maizel A.; Higgins C. P. Diffusion of perfluoroalkyl acids through clay-rich soil. J. Contaminant Hydrol. 2021, 241, 103814 10.1016/j.jconhyd.2021.103814. 33901839

[ref13] Deen W. M. Hindered transport of large molecules in liquid-filled pores. AIChE J. 1987, 33, 1409–1425. 10.1002/aic.690330902.

[ref14] Li J.; Cantwell F. F. Intra-particle sorption rate and liquid chromatographic bandbroadening in porous polymer packings III. Diffusion in the polymer matrix as the cause of slow sorption. J. Chromatogr. A 1996, 726, 37–44. 10.1016/0021-9673(95)01016-5.

[ref15] Skaug M. J.; Schwartz D. K. Tracking nanoparticle diffusion in porous filtration media. Ind. Eng. Chem. Res. 2015, 54, 4414–4419. 10.1021/ie503895b.

[ref16] Hlushkou D.; Svidrytski A.; Tallarek U. Tracer-size-dependent pore space accessibility and long-time diffusion coefficient in amorphous, mesoporous silica. J. Phys. Chem. C 2017, 121, 8416–8426. 10.1021/acs.jpcc.7b00264.

[ref17] Havlin S.; Ben-Avraham D. Diffusion in disordered media. Adv. Phys. 2002, 51, 187–292. 10.1080/00018730110116353.

[ref18] Bouchaud J. P.; Georges A. Anomalous diffusion in disordered media: Statistical mechanisms, models and physical applications. Phys. Rep. 1990, 195, 127–293. 10.1016/0370-1573(90)90099-N.

[ref19] Metzler R.; Jeon J.-H.; Cherstvy A. G.; Barkai E. Anomalous diffusion models and their properties: non-stationarity, non-ergodicity, and ageing at the centenary of single particle tracking. Phys. Chem. Chem. Phys. 2014, 16, 24128–24164. 10.1039/C4CP03465A. 25297814

[ref20] Oliveira F. A.; Ferreira R. M. S.; Lapas L. C.; Vainstein M. H. Anomalous diffusion: a basic mechanism for the evolution of inhomogeneous systems. Front. Phys. 2019, 7, 18 10.3389/fphy.2019.00018.

[ref21] Levesque M.; Bénichou O.; Rotenberg B. Molecular diffusion between walls with adsorption and desorption. J. Chem. Phys. 2013, 138, 034107 10.1063/1.4775742. 23343268

[ref22] Santamaría-Holek I.; Grzywna Z. J.; Rubi J. M. Entropic effects in diffusion-adsorption processes in micropores. Eur. Phys. J. Special Topics 2013, 222, 129–141. 10.1140/epjst/e2013-01831-2.

[ref23] Ocampo-Perez R.; Abdel daiem M. M.; Rivera-Utrilla J.; Mendez-Diaz J. D.; Sanchez-Polo M. Modeling adsorption rate of organic micropollutants present in landfill leachates onto granular activated carbon. J. Colloid Interface Sci. 2012, 385, 174–182. 10.1016/j.jcis.2012.07.004. 22858399

[ref24] Guimarães V. G.; Ribeiro H. V.; Li Q.; Evangelista L. R.; Lenzi E. K.; Zola R. S. Unusual diffusing regimes caused by different adsorbing surfaces. Soft Matter 2015, 11, 1658–1666. 10.1039/C5SM00151J. 25633342

[ref25] Koltun A. P. S.; Lenzi E. K.; Lenzi M. K.; Zola R. S. Diffusion in heterogenous media and sorption-desorption processes. Fractal Fract 2021, 5, 183 10.3390/fractalfract5040183.

[ref26] Kulasinski K.; Guyer R.; Derome D.; Carmeliet J. Water diffusion in amorphous hydrophilic systems: a stop and go process. Langmuir 2015, 31, 10843–10849. 10.1021/acs.langmuir.5b03122. 26390260

[ref27] Weisz P. B. Sorption-diffusion in heterogeneous systems Part 1. - general sorption behaviour and criteria. Trans. Farad. Soc. 1967, 63, 1801–1806. 10.1039/TF9676301801.

[ref28] Tozzi E. J.; Lavenson D. M.; McCarthy M. J.; Powell R. L. Magnetic resonance imaging to measure concentration profiles of solutes diffusing in stagnant beds of cellulosic fibers. AIChE J. 2012, 58, 59–68. 10.1002/aic.12578.

[ref29] Martinez Casillas D. C.; Longinotti M. P.; Bruno M. M.; Vaca Chavez F.; Acosta R. H.; Corti H. R. Diffusion of water and electrolytes in mesoporous silica with a wide range of pore sizes. J. Phys. Chem. C 2018, 122, 3638–3647. 10.1021/acs.jpcc.7b11555.

[ref30] Linck L. G.; Maldonado Ochoa S. A.; Ceolin M.; Corti H.; Monti G. A.; Chavez F. V.; Acosta R. H. Limits imposed by liquid/surface interactions in the determination of tortuosity in mesopores. Microporous Mesoporous Mater. 2020, 305, 110351 10.1016/j.micromeso.2020.110351.

[ref31] Chen H.-T.; Huh S.; Wiench J. W.; Pruski M.; Lin V. S.-Y. Dialkylaminopyridine-functionalized mesoporous Silica nanosphere as an efficient and highly stable heterogeneous nucleophilic catalyst. J. Am. Chem. Soc. 2005, 127, 13305–13311. 10.1021/ja0524898. 16173762

[ref32] Zhao Y. X.; Ding M. Y.; Chen D. P. Adsorption properties of mesoporous silicas for organic pollutants in water. Anal. Chim. Acta 2005, 542, 193–198. 10.1016/j.aca.2005.04.005.

[ref33] Trewyn B. G.; Nieweg J. A.; Zhao Y.; Lin V. S.-Y. Biocompatible mesoporous silica nanoparticles with different morphologies for animal cell membrane penetration. Chem. Eng. J. 2008, 137, 23–29. 10.1016/j.cej.2007.09.045.

[ref34] Mitran R. A.; Berger D.; Munteanu C.; Matei C. Evaluation of different mesoporous silica supports for energy storage in shape-stabilized phase change materials with dual thermal responses. J. Phys. Chem. C 2015, 119, 15177–15184. 10.1021/acs.jpcc.5b02608.

[ref35] Bourg I. C.; Steefel C. I. Molecular dynamics simulations of water structure and diffusion in silica nanopores. J. Phys. Chem. C 2012, 116, 11556–11564. 10.1021/jp301299a.

[ref36] Diallo S. O. Pore-size dependence and characteristics of water diffusion in slitlike micropores. Phys. Rev. E 2015, 92, 012312 10.1103/PhysRevE.92.012312. 26274167

[ref37] Olivares C.; Aarão Reis F. D. A. Interplay of adsorption and surface mobility in tracer diffusion in porous media. Phys. Rev. E 2019, 100, 022120 10.1103/PhysRevE.100.022120. 31574766

[ref38] Cai L.-H.; Panyukov S.; Rubinstein M. Mobility of nonsticky nanoparticles in polymer liquids. Macromolecules 2011, 44, 7853–7863. 10.1021/ma201583q. 22058573 PMC3205984

[ref39] Mirbagheri M.; Hill R. J. Diffusion in sphere and spherical-cavity arrays with interacting gas and surface phases. Chem. Eng. Sci. 2017, 160, 419–427. 10.1016/j.ces.2016.11.044.

[ref40] Wernert V.; Nguyen K. L.; Levitz P.; Coasne B.; Denoyel R. Impact of surface diffusion on transport through porous materials. J. Chromatogr. A 2022, 1665, 462823 10.1016/j.chroma.2022.462823. 35066296

[ref41] Miyagawa A.; Nagatomo S.; Kuno H.; Terada T.; Nakatani K. Pore size dependence of mass transfer of zinc myoglobin in a single mesoporous silica particle. Langmuir 2023, 39, 11329–11336. 10.1021/acs.langmuir.3c01017. 37523758

[ref42] http://www.fujisilysia.com/products/cariact/.

[ref43] Chevallier-Boutell I. J.; Monti G. A.; Corti H.; Olmos-Asar J. A.; Franzoni M. B.; Acosta R. H. Non-negligible interactions of alkanes with silica mesopores affect self-diffusivity: insights from first-principles calculations. Microporous Mesoporous Mater. 2021, 326, 111315 10.1016/j.micromeso.2021.111315.

[ref44] Shannon R. D. Revised effective ionic radii and systematic studies of interatomic distances in halides and chalcogenides. Acta Crystallogr., Sect. A 1976, 32, 751–767. 10.1107/S0567739476001551.

[ref45] D’Agostino C.; Mitchell J.; Gladden L. F.; Mantle M. D. Hydrogen bonding network disruption in mesoporous catalyst supports probed by PFG-NMR diffusometry and NMR relaxometry. J. Phys. Chem. C 2012, 116, 8975–8982. 10.1021/jp2123295.

[ref46] D’Agostino C.; Mitchell J.; Mantle M. D.; Gladden L. F. Interpretation of NMR relaxation as a tool for characterising the adsorption strength of liquids inside porous materials. Chem. Eur. J. 2014, 20, 13009–13015. 10.1002/chem.201403139. 25146237 PMC4510707

[ref47] Cadar C.; Ardelean I. Surface influence on the rotational and translational dynamics of molecules confined inside a mesoporous carbon xerogel. Magn. Reson. Chem. 2019, 57, 829–835. 10.1002/mrc.4819. 30577076

[ref48] He J.; Ju Y.; Lammers L.; Kulasinski K.; Zheng L. Tortuosity of kerogen pore structure to gas diffusion at molecular- and nano-scales: A molecular dynamics simulation. Chem. Eng. Sci. 2020, 215, 115460 10.1016/j.ces.2019.115460.

[ref49] Bhatia S. K.; Nicholson D. Some pitfalls in the use of the Knudsen equation in modelling diffusion in nanoporous materials. Chem. Eng. Sci. 2011, 66, 284–293. 10.1016/j.ces.2010.10.038.

[ref50] Aarão Reis F. D. A.; di Caprio D. Crossover from anomalous to normal diffusion in porous media. Phys. Rev. E 2014, 89, 062126 10.1103/PhysRevE.89.062126. 25019744

[ref51] Babayekhorasani F.; Dunstan D. E.; Krishnamoorti R.; Conrad J. C. Nanoparticle diffusion in crowded and confined media. Soft Matter 2016, 12, 8407–8416. 10.1039/C6SM01543C. 27714348

[ref52] Tallarek U.; Hlushkou D.; Rybka J.; Höltzel A. Multiscale simulation of diffusion in porous media: From interfacial dynamics to hierarchical porosity. J. Phys. Chem. C 2019, 123, 15099–15112. 10.1021/acs.jpcc.9b03250.

[ref53] Dagdug L.; Berezhkovskii A. M.; Zitserman V. Y.; Bezrukov S. M. Effective diffusivity of a Brownian particle in a two-dimensional periodic channel of abruptly alternating width. Phys. Rev. E 2021, 103, 062106 10.1103/PhysRevE.103.062106. 34271681 PMC9006170

